# Misperception of diet quality among US adults: implications for cardiometabolic health promotion

**DOI:** 10.1038/s41430-025-01605-1

**Published:** 2025-04-02

**Authors:** Eileen Lee, Nurgül Fitzgerald, Shauna Downs, Nathaniel Kuhrt, Aayush Visaria, Aparna Kalbag, Soko Setoguchi

**Affiliations:** 1 Department of Medicine, Robert Wood Johnson Medical School, Rutgers University, New Brunswick, NJ USA; 2 Department of Medicine, Yale New Haven Hospital, New Haven, CT USA; 3 Department of Nutritional Sciences, School of Environmental and Biological Sciences, Rutgers University, New Brunswick, NJ USA; 4 Department of Health Behavior, Society and Policy, School of Public Health, Rutgers University, Newark, NJ USA; 5 New Jersey Medical School, Rutgers University, Newark, NJ USA; 6 Department of Medicine, Thomas Jefferson University Hospital, Philadelphia, PA USA; 7 Rutgers Health- RWJBarnabas Health Center for Climate, Health, and Healthcare, Institute for Health, Health Care Policy and Aging Research, Rutgers University, New Brunswick, NJ USA; 8 Department of Epidemiology and Biostatistics, School of Public Health, Rutgers University, Piscataway, NJ USA

**Keywords:** Nutrition, Epidemiology

## Abstract

**Background:**

Diet is pivotal in preventing and managing cardiometabolic diseases. Our study aimed to describe the prevalence of poor diet quality and perceiving a poor diet as healthy and to determine individual-level factors associated with these groups.

**Methods:**

This cross-sectional study analyzed seven 2-year cycles of National Health and Nutrition Examination Survey (NHANES) data from 2005 to 2018, which included non-pregnant adults between 20 and 85 years old, who completed a one-day 24-h dietary recall and dietary interview. Diet quality was measured using the American Heart Association (AHA) primary diet score, and perceived diet quality was based on NHANES questionnaire response.

**Results:**

Among 31,644 adults, the prevalence of poor diet quality was 47%. Male sex and smoking were associated with a higher risk of poor diet quality, while older age, higher levels of education, increased income, diabetes mellitus diagnosis, and increased vigorous activity levels were associated with a lower risk of poor diet quality. Among adults with poor diet quality (*n* = 14,952), 23% perceived their diet as healthy. In multivariable analysis, older age, higher education, and vigorous activity level were associated with a higher risk of perceiving a poor diet as healthy.

**Conclusions:**

Nearly half of US adults had poor diet quality based on AHA guidelines for cardiovascular health, yet nearly a quarter of them perceived their diet as healthy. This gap underscores the need for focused educational efforts and interventions in both healthcare and public health settings to dispel diet-related misperceptions and motivate the adoption of a healthier diet to address cardiometabolic health.

## Introduction

The prevalence of cardiometabolic diseases (CMD) including chronic conditions such as diabetes [1], hypertension [2, 3], obesity [4, 5], and cardiovascular disease (CVD) [6] as well as multimorbidity with coexistence of CMD, has largely increased between 1999 and 2018 [7]. A healthy diet plays an important role in preventing [8, 9] and managing [10] CMD. Generally, heart-healthy dietary patterns associated with low CVD risk contain primarily fruits and vegetables, foods made with whole grains, healthy sources of protein (plants, fish and seafood, low-fat or fat-free dairy products, and lean cuts and unprocessed forms of meat or poultry), liquid plant oils, and minimally processed foods [11]. These dietary patterns, including the Mediterranean or Dietary Approaches to Stop Hypertension (DASH) diets, also contain fewer added sugars and less salt [10, 12].

Despite public health efforts to improve the cardiometabolic health of the US population, diet quality scores have been consistently lower than desired goals [10]. For example, guidelines from the American Heart Association (AHA) Life’s Essential 8, including core health behaviors (smoking, physical activity, diet, and weight) and health factors (cholesterol, blood pressure, and glucose control), identifies diet among the metrics with the lowest scores [10]. A recent cross-sectional analysis from the National Health and Nutrition Examination Survey (NHANES) 2013–2018 cycles found diet quality scores to range from 23.8 to 47.7 out of 100 [13]. Prior studies have consistently demonstrated poor diet quality among Americans, including a separate NHANES analysis of 1999–2010 survey cycles [14].

Inaccurate perceptions of healthy diet have been reported as a barrier to promoting healthy dietary patterns [15]. A small number of studies in various populations, including adults seeking weight loss and a nationally representative sample in Mexico, suggested that perceived diet quality may differ from measured diet quality [16–21]. However, differences between objective and perceived diet quality as well as risk factors for this discordance have yet to be assessed in a sample representative of the US population. Thus, we aimed to (1) determine the prevalence of poor diet quality and its associated individual-level factors and (2) investigate the prevalence of perceiving poor diet quality as healthy along with its associated factors in a contemporary US representative sample.

## Methods

### Data source

NHANES is a repeated cross-sectional investigation of nationally representative samples of adults and children in the US. The sample design consists of multiyear, stratified, clustered four-stage samples to ensure representation across all 50 states and the District of Columbia. To achieve sufficient sample size in subgroups of the general population such as age, race and Hispanic origin, sex, income status, and geographical location, some minority subgroups are oversampled relative to the general sampling rate. Survey weights are then provided to produce nationally representative estimates for the entire population. Additional details on study design and operation may be found elsewhere [22]. It combines interviews, physical examinations, and laboratory tests to assess demographic, socioeconomic, dietary, and health-related information about participants [23]. The NHANES Dietary Interview Component includes a 24-h dietary recall to assess detailed dietary intake consumed during the 24-h period prior to the interview (midnight to midnight) along with a follow-up dietary recall interview collected 3–10 days later; due to decreased sample size in the secondary dietary recall, only the first single day 24-h dietary recall is included in this study.

### Diet quality measures

We estimated the AHA Diet Score derived from the AHA 2020 Strategic Impact Goals using a single day 24-h dietary recall [24, 25]. The diet score includes 8 dietary components. Each dietary component was scored between 0 and 10 (eTable 1). The five primary dietary metrics were fruits and vegetables (consumption range: 0 to ≥4.5 cups/day), fish and shellfish (0 to ≥7 oz/week), sodium (≤1500 to ≥4500 mg/day), sugar-sweetened beverages (SSBs) (≤36 to >210 fl oz/week), and whole grains (0 to ≥3 oz/day). Secondary dietary metrics were nuts, seeds, and legumes (0 to ≥4 servings/day), processed meats (≤3.5 to >17.5 oz/week), and saturated fat (≤7 to >15% of energy calculated in calories). Higher consumption of 4 dietary components considered to be beneficial foods – fruits and vegetables, fish and shellfish, whole grains, nuts, seeds, and legumes – denoted a higher score. Conversely, higher consumption of 4 dietary components considered to be harmful foods – sodium, SSBs, processed meats, and saturated fat – resulted in a lower score.

To generate the diet score from NHANES, we utilized the USDA Food Patterns Equivalents Database (FPED), which converts food and beverages reported during the NHANES dietary interview into 37 USDA food pattern components aligned with the AHA diet score metrics [26].

The AHA primary diet score ranged from 0 (worst) to 50 (best), while the secondary diet score ranged from 0 (worst) to 80 (best). To allow for comparisons, both scores were then rescaled to a range of 0–100%, generating a standardized score [27]. Diet quality was categorized into three groups based on AHA guidelines: poor diet score <40%, intermediate from 40% to <80%, and ideal diet $\ge$80% [25].

### Perceived diet quality

We assessed perceived diet quality based on the survey question: “How healthy is your overall diet?”, found in the Diet Behavior and Nutrition section of the NHANES interview. Responses included excellent, very good, good, fair, and poor. Excellent or very good responses corresponded to healthy perceived diet quality.

### Study population

The primary study population included adults aged 20 years and older across 7 consecutive 2-year cycles of NHANES from 2005–2006 through 2017–2018. Participants 85 years and older were coded as 85 years of age. We included participants who completed the Diet Behavior and Nutrition section of the interview and provided a response to the first 24-h dietary recall interview. We excluded those without recorded FPED data and pregnant women. We also identified a subgroup of the primary study population who had poor diet quality (AHA primary score <40%) and responded to the perceived diet quality question.

### Sociodemographic and Other Characteristics

We identified sociodemographic factors including age, sex, race, and ethnicity (non-Hispanic White, non-Hispanic Black, Hispanic [including Mexican American and Other Hispanic], or other [including non-Hispanic Asian and multiracial participants]), education level (less than high school, high school, some college, college graduate or above), household income (<$20K, $20–75K, $75K+), language of interview (English or Spanish), and poverty income ratio calculated by dividing family or individual income by the Department of Health and Human Services poverty guidelines. We also examined self-reported comorbidities recorded in NHANES to identify congestive heart failure, coronary heart disease, myocardial infarction, stroke, emphysema, cancer, diabetes mellitus, hypertension, and high cholesterol. Depression was measured using the Patient Health Questionnaire-9 (PHQ-9). In addition to self-reporting, comorbidities were also identified using the Prescription Medications section of the interview data. For example, participants taking anti-diabetes medications without having self-reported diabetes were considered to have diabetes. Additional measurements and lifestyle-related factors included body mass index (BMI), waist circumference (cm), triglyceride levels, number of alcoholic drinks per day, binary physical activity variables (moderate: >10 min of activity causing light sweating or a slight to moderate increase in breathing or heart rate in the last 30 days; vigorous: >10 min of activity causing heavy sweating or large increases in breathing or heart rate), a binary smoking variable (≥100 cigarettes in lifetime, <100 cigarettes), and a binary variable representing awareness of US dietary guidelines including any of the following: Dietary Guidelines for Americans, MyPyramid, or MyPlate. Height, weight, and waist circumference were measured from survey participants by trained health technicians. BMI was calculated using measured height and weight. Triglyceride levels were recorded from laboratory samples of participants.

### Statistical analysis

To account for the complex survey design in NHANES, all analyses utilized sampling weight, strata, and primary sampling unit parameters to match the US civilian noninstitutionalized resident population [22]. We first performed a descriptive analysis of sociodemographic, comorbidity, and lifestyle characteristics among all study participants in the primary study population who met inclusion and exclusion criteria grouped by ideal/intermediate vs. poor diet quality. We also summarized responses of perceived diet quality to determine the discrepancy between assessed and perceived diet quality. To identify factors associated with poor diet quality, we used a survey-weighted univariate and multivariable Poisson regression with robust error estimation [28] including age (20–34, 35–49, 50–64, and 65+ years old), sex, race/ethnicity, education, household income, comorbidities (myocardial infarction, diabetes mellitus, hypertension, high cholesterol), waist circumference (low risk: <102 cm, <88 cm and high risk ≥102 cm, ≥88 cm for men and women, respectively) [29], BMI (underweight <18.5, healthy weight 18.5 to <25, overweight 25.0 to <30, and obesity ≥30.0) [30], alcoholic drinks (moderate: ≤1 drink per day in women, ≤2 drinks per day in men), vigorous activity, and smoking. For the multivariable regression models, each predictor was adjusted for all remaining covariates.

As subgroup analyses in the secondary group, we performed a descriptive analysis of participant characteristics across perceived diet quality responses of non-healthy (Poor, Fair, and Good) and healthy (Very Good, and Excellent). We used a similar survey-weighted Poisson regression approach as the overall cohort to predict the risk of perceiving a poor diet quality as healthy. In a sensitivity analysis among this subgroup, we reclassified a perceived diet quality response of “good” as healthy perceived diet quality (Good, Very Good, and Excellent) and non-healthy (Poor and Fair). Statistical significance was defined as *p* < 0.05. We used R Studio, version 4.2.2 (R Group for Statistical Computing) for all analyses.

## Results

### Participant characteristics

**Fig. 1:**
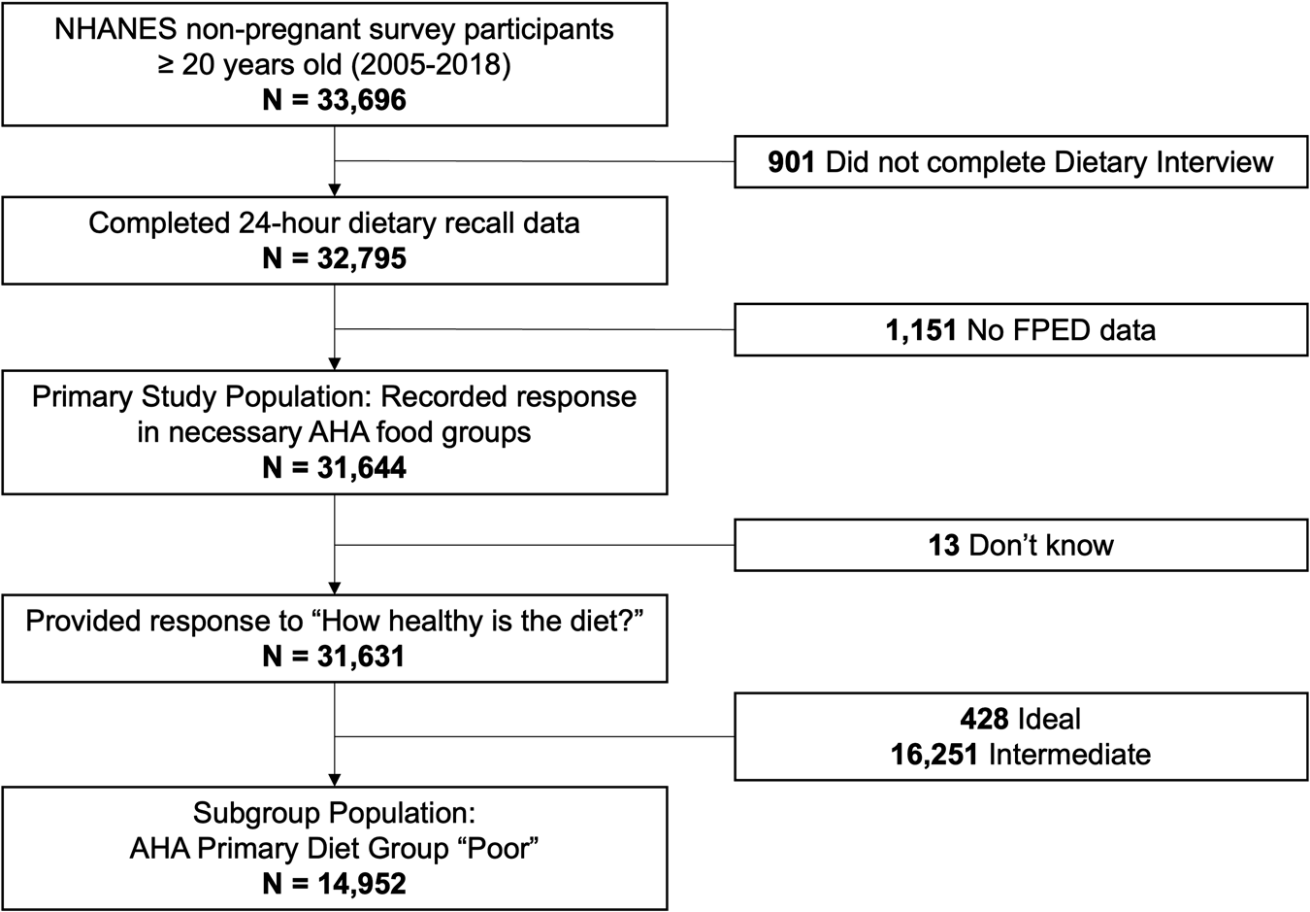
Selection criteria for adult participants in study population. NHANES National Health and Nutrition Examination Survey, AHA American Heart Association, FPED Food Patterns Equivalents Database used to convert foods and beverages from dietary recall responses in NHANES to AHA Diet Score.

The study included 31,644 adults (15,811 women [51%]; 13,652 Non-Hispanic White [68%]; mean [SD] age 48 [17] years) who completed a 24-h dietary recall (Fig. 1, Table 1) and met inclusion/exclusion criteria. Over 60% of participants had at least some college education. High cholesterol (41%) and hypertension (32%) were the most prevalent comorbidities. The average BMI was 29 (SD: 7). Nearly 50% of participants reported engaging in moderate physical activities; 28% of participants reported engaging in vigorous activities. Of the study population who provided a response, 35% of participants noted hearing about US dietary guidelines, including 32% who had poor diet quality.

**Table 1:** Demographic characteristics, comorbidities, and lifestyle factors based on AHA Primary Diet Score Groups among adult participants in NHANES 2005–2018.

	Missing (*n*)	Overall	Ideal & Intermediate	Poor
Total population (*n*)^a^		31,644	16,684	14,960
Sociodemographics				
Age	0	48 (17)	50 (17)	45 (16)
Sex	0			
Female		15,811 (51%)	8749 (54%)	7062 (47%)
Male		15,833 (49%)	7935 (46%)	7898 (53%)
Race/Ethnicity	0			
Non-Hispanic White		13,658 (68%)	7307 (69%)	6351 (66%)
Hispanic		7961 (14%)	3963 (13%)	3998 (15%)
Non-Hispanic Black		6716 (11%)	3401 (10%)	3315 (12%)
Other		3309 (7.6%)	2013 (8.6%)	1296 (6.5%)
Education	23			
Less than High school		7709 (16%)	3777 (14%)	3932 (18%)
High school graduate		7274 (23%)	3567 (21%)	3707 (26%)
Some college		9367 (32%)	4775 (31%)	4592 (33%)
College graduate or above		7271 (29%)	4551 (35%)	2720 (23%)
Household income	1523			
Less than $20,000		6310 (15%)	3094 (13%)	3216 (16%)
$20,000-$75,000		15,977 (50%)	8289 (48%)	7688 (51%)
Greater than $75,000		7834 (36%)	4505 (38%)	3329 (33%)
Interview language	0			
English		27,653 (94%)	14,717 (95%)	12,936 (93%)
Spanish		3990 (6.0%)	1966 (5.4%)	2024 (6.7%)
Poverty income ratio	2633	3.01 (1.65)	3.16 (1.64)	2.84 (1.65)
Comorbidities				
Congestive heart failure	0	1022 (2.4%)	613 (2.8%)	409 (1.9%)
Coronary artery disease	0	1319 (3.4%)	811 (4.2%)	508 (2.6%)
Myocardial infarction	0	1359 (3.3%)	791 (3.7%)	568 (2.8%)
Stroke	0	1216 (2.9%)	680 (3.1%)	536 (2.7%)
Emphysema	37	649 (1.8%)	310 (1.6%)	339 (1.9%)
Cancer	24	3053 (10%)	1853 (12%)	1200 (8.5%)
Diabetes mellitus	602	4426 (10%)	2736 (12%)	1690 (8.1%)
Hypertension	49	11,416 (32%)	6455 (34%)	4961 (30%)
High cholesterol	4128	11,842 (41%)	6847 (44%)	4995 (37%)
Depression	14,704	4186 (24%)	1961 (22%)	2225 (26%)
BMI (kg/m^2^)	291	29 (7)	29 (6)	29 (7)
Waist circumference (cm)	984	99 (16)	98 (16)	100 (17)
Triglyceride (mg/dL)	1629	154 (115)	149 (109)	159 (121)
Lifestyle				
Alcoholic drinks per day	0	0.24 (0.66)	0.26 (0.69)	0.22 (0.63)
Moderate activity	99	13,267 (48%)	7600 (52%)	5667 (44%)
Vigorous activity	141	7403 (28%)	4063 (30%)	3340 (26%)
Smoking	15	14,251 (45%)	6930 (42%)	7321 (49%)
Dietary guidelines awareness	10,135	6109 (35%)	3353 (38%)	2756 (32%)
AHA primary score (standardized)	0	42 (16)	54 (11)	28 (8)
AHA secondary score (standardized)	0	44 (15)	53 (13)	34 (11)
Perceived diet quality	13			
Poor		1880 (5.4%)	766 (4.0%)	1114 (7.0%)
Fair		7530 (21%)	3312 (17%)	4218 (26%)
Good		12,957 (42%)	6742 (41%)	6215 (43%)
Very good		6549 (23%)	4085 (28%)	2464 (17%)
Excellent		2715 (8.5%)	1774 (11%)	941 (6.0%)

*BMI* body mass index, *AHA* American Heart Association

^a^Categorical variables are reported with unweighted counts and survey-weighted percentages. Continuous variables are reported with mean and standard deviation.

**Fig. 2:**
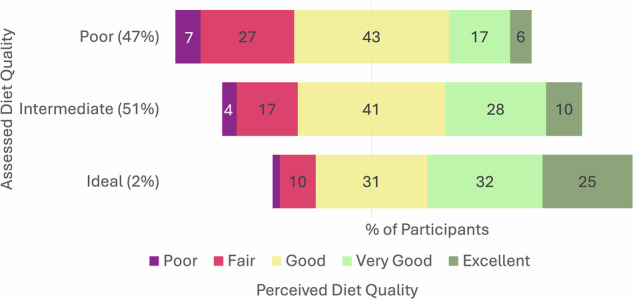
Perceived vs. assessed diet quality among adult participants. Poor assessed diet quality defined as scaled AHA diet score <40%, intermediate defined as ≥40% and <80%, ideal defined as ≥80%. Perceived diet quality based on NHANES survey question response.

Among all participants, average standardized AHA primary and secondary scores were 42 and 44 (out of 100), respectively. Overall, 47% of participants had poor measured diet quality, 51% had intermediate measured diet quality, and 1.4% had ideal measured diet quality (Fig. 2). Regardless of measured diet quality, the most common survey response for perceived diet quality was “good” (42%). A large proportion of participants (31%) also perceived their diet as “healthy”. Among those with poor diet quality, 23% of participants reported a healthy perceived diet quality.

### Predictors of poor measured diet quality

Table 2 displays survey-weighted univariate and multivariable regression analysis results of factors associated with poor diet quality. Within univariate analyses, male sex (reference: women), non-Hispanic Black and Hispanic (reference: non-Hispanic White), high-risk waist circumference (reference: low risk), overweight BMI (reference: normal BMI), and smoking (versus not smoking) were significantly associated with poor diet (Risk Ratio [RR] ranging from 1.11 to 1.17). Older age, both 50–65 years and 65+ years (reference: 20–34 years), “Other” race (versus non-Hispanic White), higher levels of education, increases in household income, having a history of myocardial infarction, diabetes mellitus, or hypertension, and vigorous activity were associated with a decreased likelihood of poor diet (RR ranging from 0.63 to 0.91).

**Table 2:** Relative risk of poor objective diet quality among adult participants.

	Univariate	Multivariable
Variable	RR (95% CI)	RR (95% CI)
Age group^a^		
35–49	0.95 (0.91, 1.00)	**0.94 (0.90, 0.99)**
50–64	**0.81 (0.77, 0.85)**	**0.79 (0.74, 0.83)**
65+	**0.63 (0.59, 0.67)**	**0.61 (0.56, 0.66)**
Sex^b^		
Male	**1.15 (1.12, 1.19)**	**1.15 (1.11, 1.20)**
Race/Ethnicity^c^		
Hispanic	**1.13 (1.08, 1.18)**	1.00 (0.95, 1.05)
Non-Hispanic Black	**1.11 (1.06, 1.16)**	1.00 (0.96, 1.05)
Other	**0.88 (0.81, 0.94)**	**0.88 (0.81, 0.94)**
Education^d^	**0.78 (0.75, 0.81)**	**0.81 (0.77, 0.86)**
Household income^e^	**0.89 (0.86, 0.92)**	**0.94 (0.90, 0.98)**
Comorbidities		
Myocardial infarction	**0.85 (0.77, 0.94)**	0.95 (0.85, 1.06)
Diabetes mellitus	**0.90 (0.86, 0.93)**	**0.83 (0.77, 0.88)**
Hypertension	**0.87 (0.84, 0.90)**	1.00 (0.96, 1.05)
High cholesterol	0.98 (0.95, 1.01)	1.00 (0.96, 1.04)
High-risk waist circumference^f^	**1.09 (1.05, 1.14)**	1.01 (0.95, 1.07)
BMI^g^		
Obesity	1.02 (0.97, 1.06)	1.07 (1.00, 1.15)
Overweight	**1.17 (1.01, 1.34)**	1.02 (0.96, 1.07)
Underweight	0.94 (0.86, 1.02)	1.09 (0.93, 1.29)
Above moderate alcoholic drinks per day^h^	**0.84 (0.80, 0.87)**	0.96 (0.88, 1.05)
Vigorous activity^i^	**0.91 (0.88, 0.95)**	**0.87 (0.83, 0.92)**
Smoking^j^	**1.15 (1.11, 1.19)**	**1.09 (1.05, 1.13)**

^a^Reference range: Age group 20–34; ^b^Reference range: Female; ^c^Reference range: White and Other includes non-Hispanic Asian and multiracial; ^d^Ordered variable: Less than High School, High school graduate, Some college, College graduate or above; ^e^Ordered variable: Less than $20K, $20–75K, $75K+; ^f^Reference range: Low-risk waist circumference; ^g^Reference range: Healthy; ^h^Reference range: Moderate alcoholic drinks per day; ^i^At least 10 continuous minutes; ^j^At least 100 cigarettes in lifetime

Statistically significant values are in bold

In multivariable regression, male sex and smoking were significantly associated with a 15% and 9% increase in risk of a poor diet, respectively. Several factors were associated with a decreased risk of poor diet quality. Older age, increasing household income, and higher levels of education along with non-Hispanic Asian and multiracial ethnicities remained significantly associated with a decreased likelihood of a poor diet (RR ranging from 0.61 to 0.94). Participants with diabetes mellitus had a 17% reduction in risk of poor diet quality compared to those without the diagnosis. Those who engaged in vigorous activity also had a 13% reduction in risk of poor diet quality.

### Subgroup participant characteristics

Among 14,952 participants considered to have poor diet quality, demographics remained similar to the larger study population (eTable 2). The “Overall” category in eTable 2 matches the “Poor” group in Table 1. While 44% of participants reported moderate activity in the last 30 days, fewer reported vigorous activity (26%) consistent with patterns in the overall sample. Approximately 32% of participants had heard of dietary guidelines, including 34% of those who perceived their poor diet quality as healthy. Participants with poor diet quality had mean AHA standardized primary and secondary scores of 28 (out of 100) and 34, respectively. We found that 23% of participants perceived their poor diet quality as healthy (i.e., answered “excellent” or “very good”), 43% perceived their poor diet as good, and 34% were aligned in their perceived diet quality with a rating of poor or fair.

### Predictors of perceiving poor diet quality as healthy

Among those with poor diet quality, several characteristics, health conditions, and lifestyle factors were associated with perceiving poor diet quality as healthy (Table 3). In univariate analyses, increasing age, levels of education, and household income were positively related to perceiving poor diet quality as healthy. Non-Hispanic Asian and multiracial group (reference: non-Hispanic White), myocardial infarction history, above moderate alcoholic consumption per day, and vigorous activity also indicated a significantly higher likelihood of perceiving their poor diet quality as healthy (RR ranging from 1.18 to 1.97). Non-Hispanic Black and Hispanic race/ethnicity (reference: non-Hispanic White), having diabetes mellitus, high-risk waist circumference, BMI (obesity, overweight, underweight versus normal BMI), and smoking (versus not smoking) were all associated with a significantly decreased likelihood of perceiving the diet as healthy (RR ranging from 0.53 to 0.91).

**Table 3:** Risk ratios of perceiving poor diet quality as healthy among subgroup population.

	Univariate	Multivariable
Variable	Risk Ratio (95% CI)	Risk Ratio (95% CI)
Age group^a^		
35–49	**1.18 (1.05, 1.32)**	**1.35 (1.20, 1.53)**
50–64	**1.48 (1.30, 1.69)**	**1.92 (1.66, 2.22)**
65+	**1.97 (1.76, 2.19)**	**2.59 (2.22, 3.02)**
Sex^b^		
Male	0.99 (0.92, 1.08)	0.93 (0.84, 1.03)
Race/Ethnicity^c^		
Hispanic	**0.63 (0.56, 0.72)**	**0.75 (0.65, 0.88)**
Non-Hispanic Black	**0.78 (0.71, 0.86)**	**0.87 (0.78, 0.97)**
Other	**1.23 (1.08, 1.40)**	1.15 (0.98, 1.34)
Education^d^	**1.44 (1.31, 1.58)**	**1.25 (1.11, 1.40)**
Household income^e^	**1.20 (1.10, 1.30)**	0.98 (0.90, 1.08)
Comorbidities		
Myocardial infarction	**1.21 (1.01, 1.44)**	1.11 (0.88, 1.39)
Diabetes mellitus	**0.78 (0.66, 0.92)**	0.85 (0.70, 1.02)
Hypertension	0.93 (0.87, 1.00)	**0.87 (0.79, 0.97)**
High cholesterol	1.03 (0.94, 1.13)	0.93 (0.83, 1.03)
High-risk waist circumference^f^	**0.67 (0.61, 0.74)**	**0.80 (0.69, 0.93)**
BMI^g^		
Obesity	**0.53 (0.47, 0.59)**	**0.69 (0.58, 0.81)**
Overweight	**0.86 (0.78, 0.95)**	0.93 (0.82, 1.06)
Underweight	0.88 (0.63, 1.21)	0.91 (0.63, 1.30)
Above moderate alcoholic drinks per day^h^	**1.24 (1.05, 1.47)**	1.15 (0.95, 1.39)
Vigorous activity^i^	**1.45 (1.33, 1.57)**	**1.50 (1.36, 1.66)**
Smoking^j^	**0.91 (0.84, 0.98)**	0.96 (0.86, 1.06)

^a^Reference range: Age group 20–34; ^b^Reference range: Female; ^c^ Reference range: White and Other includes non-Hispanic Asian and multiracial; ^d^Ordered variable: Less than High School, High school graduate, Some college, College graduate or above; ^e^Ordered variable: Less than $20 K, $20-75 K, $75 K + ; ^f^Reference range: Low-risk waist circumference; ^g^Reference range: Healthy; ^h^Reference range: Moderate alcoholic drinks per day; ^i^At least 10 continuous minutes; ^j^At least 100 cigarettes in lifetime.

Statistically significant values are in bold

In multivariable analysis, increases in age and education, and vigorous activity remained significantly associated with an increased risk of perceiving poor diet quality as healthy (RR ranging from 1.25 to 2.59). Compared to participants aged 20–34 years, those aged 35–49 years, 50–64 years, and 65+ years had a 35%, 92%, and 159% increased risk of perceiving their poor diet as healthy. A 25% increased risk with higher levels of education and a 50% increased risk with vigorous activity were also found. In contrast, Hispanic or Non-Hispanic Black (reference: Non-Hispanic White), hypertension, high-risk waist circumference, and obesity BMI (reference: normal BMI) were less likely to perceive a poor diet as healthy (RR ranging from 0.69-0.87). Hispanic and Non-Hispanic Black race/ethnicity had a 25% and 13% decreased risk, respectively. Both high-risk waist circumference (20%) and obesity (31%) also resulted in decreased risk of perceiving a poor diet as healthy.

### Sensitivity analysis

As a part of planned sensitivity analyses, we modified our definition of healthy perceived diet quality to include not only “very good” and “excellent” responses, but also “good” responses, because it was possible for participants to believe that “good” equates to healthy. This change resulted in 64% of participants with poor diet perceiving it as healthy compared to 23% previously. Demographic characteristics remained similar among those with a healthy diet perception using the new definition (eTable 3). High cholesterol and hypertension remained the most prevalent comorbidities.

Risk factors associated with perceiving a poor diet as healthy also remained similar (eTable 4) with a few additional significant factors associated with perceiving a poor diet as healthy in multivariable analysis. Male sex compared to female sex and smoking compared to non-smoking had a 5% and 8% decreased risk of perceiving a poor diet as healthy, respectively. Conversely, increased household income (6%) and diabetes mellitus diagnosis (10%) had an increased risk of perceiving a poor diet as healthy.

## Discussion

This study examined the prevalence of poor diet quality among a representative sample of US adults and assessed individual-level factors associated with both poor diet quality and perceiving it as healthy. Overall, we found nearly half of US adults had poor diet quality. Male sex and smoking were associated with poor diet quality, while increased age, higher socioeconomic status (education and income), diabetes mellitus diagnosis, and vigorous activity levels were associated with lower risk. We also identified nearly a quarter of adults with poor diet quality perceive it as healthy. Despite being associated with healthier measured diet quality scores, older age, higher education, and higher vigorous activity were associated with a higher risk of perceiving a poor diet as healthy.

This is the largest study analyzing diet quality and perceived diet quality in the US. A prior analysis of NHANES data from 1999 to 2010 identified improvement in diet quality over time, though overall diet quality remained poor [14], and a separate study concluded nearly the entire US population’s diet does not meet federal dietary recommendations [31]. Consistent with these prior studies, we identified a large proportion of Americans consume a diet poor in quality. When assessing demographic and socioeconomic factors associated with poor diet quality, we found older age, female sex, higher education, and higher income to be associated with a lower likelihood of poor diet quality. This aligns with prior studies [14, 32] and could stem from older adults becoming more conscious of their health as they age, having more access to and better use of dietary resources, and improving diets to prevent or manage chronic disease.

Expanding on the current literature, we identified medical conditions such as diabetes mellitus and lifestyle-related behaviors, including vigorous activity level, to have a significant positive association with diet quality. We found participants with diabetes mellitus had a 17% lower likelihood of poor diet quality, possibly secondary to increased availability and accessibility of nutrition recommendations and guidelines for individuals with chronic conditions [33–35]. Diabetes education continues to expand in the US, promoting increased awareness of lifestyle factors, including diet [36, 37]. Importantly, some level of vigorous activity in the last 30 days was also associated with a 13% less likelihood of having a poor diet. Adherence to both dietary and physical activity guidelines improves overall health and healthy aging [38, 39].

In contrast to protective factors, we found smoking to be an independent risk factor of poor diet. While one prior study found heavy smokers (smoking more than 20 cigarettes per day) to have a significantly poorer diet quality than those who never smoked, independent of socioeconomic, lifestyle, and biological factors [40], our study shared similar results with a more liberal smoker definition (smoking >100 cigarettes in a lifetime), suggesting an association between even limited smoking exposure and poor diet.

Our investigation of risk factors associated with perceiving poor diet quality as healthy is unique to our analysis. Interestingly, while older age, higher levels of education, and vigorous activity level were associated with a lower likelihood of poor measured diet quality in the overall sample, they were also associated with a higher risk of perceiving a poor diet as healthy among the subgroup of individuals with poor diet quality score. Regardless of measured diet quality, which was not assessed in Powell-Wiley et al., the study identified participants who were older, non-Hispanic White, with higher levels of education or with increased poverty income ratio were more likely to have a high perceived diet quality; however, this study did not assess physical activity level [16]. A possible explanation of our findings on vigorous activity is adults may feel exercising contributes to overall better health and may forgo making changes in their diets due to higher perceived diet quality despite eating poorly.

In the subgroup of individuals with poor diet quality, among non-Hispanic Black and Hispanic participants, there was greater concordance of objective and perceived diet quality compared to non-Hispanic Whites. This finding differs from a prior study of cancer survivors which suggested that Hispanic patients were more likely to over-rate their diet quality than non-Hispanic White patients [19]. This difference may be attributed to the non-equivalence of the target sample (cancer survivors vs. general population with poor diet quality), the small sample size of Hispanic cancer patients, and the definition of the healthy diet perception metric in the study of cancer patients where the most common over-rated perceived diet quality response was fair in the previous study, which was not considered healthy in our definition.

Our results also suggest that individuals with higher waist circumference and BMI were less likely to perceive their poor diet as healthy compared to those with smaller waist circumference and lower BMI, respectively. Prior research on older adults showed relationships between obesity, measured diet quality, and self-assessed health-related quality of life; perceived health scores were significantly lower for participants with unhealthy diets and obesity [41]. However, there is limited evidence between perceived diet quality and BMI specifically. While the inverse association between BMI and measured diet quality may be anticipated [42, 43], we also found that having a higher BMI was associated with an increased likelihood of unhealthy perceived diet quality.

Perceived diet quality is an important psychosocial factor that influences measured diet quality and is often predictive of food consumption behavior [18, 44, 45]. Those who are overly optimistic about their diet quality may unknowingly sustain poor dietary habits due to underestimation of health risks [44]. Our study found a higher risk of a disconnect between perceived and measured diet quality among adults with certain demographic, socioeconomic, and lifestyle-related characteristics: those with normal BMI, lower waist circumference, higher levels of education, older age, and vigorous exercise practice. Increasing awareness about these characteristics of the adult population with misperceptions of diet quality is clinically important to inform practice for nutrition, medical care, and public health. For example, healthcare providers may screen patients with high-risk demographics and offer targeted education and intervention to improve diet quality. Employing more nutritionists and health psychologists in primary care settings can also help patients follow through on diet-related behavior changes.

Beyond the healthcare setting, tailored education programs and social marketing campaigns about healthy eating have the potential to reduce the misalignment between perceived and measured diet quality. Specifically, programs or campaigns that highlight common misperceptions related to healthy eating, such as all dietary fats are bad for your health [46], could help address the disconnect between perceptions and objective measures. In addition to public health initiatives aimed at creating increased awareness related to healthy eating, policy changes can help to make it easier for consumers to make healthier choices or at least informed choices with the correct perception of what constitutes a healthy diet. In the US, adopting easy-to-interpret front-of-pack labeling systems, such as warning labels, could help provide consumers with more information about the quality of the food products that they purchase and consume. These types of labels have been found to be effective in terms of encouraging healthy eating [47].

Strengths of our study include a large US representative sample, standardized data aggregation, and rigorous data quality control methods to improve the generalizability of the findings. Several limitations are worth noting. First, 24-h dietary recall is self-reported and known to inaccurately estimate overall intake, often underreporting total energy intake; we also used only a single day of dietary recall [48, 49]. However, this method of dietary intake remains a low-cost, low-burden alternative and presents more accurate results when aggregated at a group or population level as in this study [48]. Perceived diet quality measurement using a single component of the questionnaire may also be subject to response bias where a truthful response may be difficult to elicit and can also fail to account for cultural differences in interpretation of the Likert scale, such as considering a response of “good” to align with a belief of eating healthy. Our study included a sensitivity analysis to assess the impact of including “good” as healthy perceived diet quality, as participants may have interpreted “good” as sufficient to support health. This resulted in 64% of participants who perceived a poor diet as healthy, showing a larger discrepancy among adults than initially found. The analysis also identified additional risk (higher household income) and protective (diabetes mellitus diagnosis and smoking history) factors for perceiving a poor diet as healthy. Our restriction to “very good” and “excellent” responses maintained higher specificity for defining a healthy perceived diet quality.

## Conclusion

Our study suggests that nearly half of US adults have poor diet quality based on dietary targets from the AHA 2020 Strategic Impact Goals, yet almost a quarter of them perceived their diet as healthy. While adults with older age or higher socioeconomic status (higher education, higher income) were less likely to have a poor diet in general, among those with poor diet quality, those with older age or higher education were more likely to perceive a poor diet as healthy. Addressing the disconnect between perceived and actual diet quality through the active involvement of healthcare providers, focused education interventions, and public health and policy initiatives may be helpful to reduce the misconception, modify eating behavior, and promote cardiometabolic health among US adults.

## Supplementary information

Supplementary Material

## Data Availability

The datasets generated during and/or analyzed during the current study are available from the corresponding author upon reasonable request.
