# Supplementary material for: Misperception of diet quality among US adults: implications for cardiometabolic health promotion
Source: Eur J Clin Nutr. 2025 Apr 2;79(8):762–9. doi: 10.1038/s41430-025-01605-1 (PMC12353818; doi:10.1038/s41430-025-01605-1)
Supplement: Supplementary file 1 — Supplementary Material [file 41430_2025_1605_MOESM1_ESM.docx]

# **Supplemental Material**

## eTable 1. AHA Dietary Targets and Healthy Diet Score for Defining Cardiovascular Health

This table shows the American Heart Association targets, consumption ranges for the Alternative Healthy Diet Score, and the Alternative Scoring range for primary diet metrics and secondary diet metrics as reported in the AHA Journal.

| Primary dietary metrics[†](https://www-ahajournals-org.proxy.libraries.rutgers.edu/reader/content/10.1161/CIR.0000000000000757/format/epub/EPUB/xhtml/index.xhtml#tab5-1fn3) | | | |
| --- | --- | --- | --- |
| Metric | AHA Target | Consumption Range | Score Range |
| Fruits and vegetables | ≥4.5 cups/d[‡](https://www-ahajournals-org.proxy.libraries.rutgers.edu/reader/content/10.1161/CIR.0000000000000757/format/epub/EPUB/xhtml/index.xhtml#tab5-1fn4) | 0 to ≥4.5 cups/d[‡](https://www-ahajournals-org.proxy.libraries.rutgers.edu/reader/content/10.1161/CIR.0000000000000757/format/epub/EPUB/xhtml/index.xhtml#tab5-1fn4) | 0–10 |
| Fish and shellfish | 2 or more 3.5-oz servings/wk (≥200 g/wk) | 0 to ≥7 oz/wk | 0–10 |
| Sodium | ≤1500 mg/d | ≤1500 to >4500 mg/d | 10–0 |
| SSBs | ≤36 fl oz/wk | ≤36 to >210 fl oz/wk | 10–0 |
| Whole grains | 3 or more 1-oz-equivalent servings/d | 0 to ≥3 oz/d | 0–10 |
| Secondary dietary metrics[†](https://www-ahajournals-org.proxy.libraries.rutgers.edu/reader/content/10.1161/CIR.0000000000000757/format/epub/EPUB/xhtml/index.xhtml#tab5-1fn3) | | | |
| Nuts, seeds, and legumes | ≥4 servings/wk (nuts/seeds: 1 oz; legumes: ½ cup) | 0 to ≥4 servings/d | 0–10 |
| Processed meats | 2 or fewer 1.75-oz servings/wk (≤100 g/wk) | ≤3.5 to >17.5 oz/wk | 10–0 |
| Saturated fat | ≤7% energy | ≤7 to >15 (% energy) | 10–0 |
| AHA Diet Score (primary) | Ideal: 4 or 5 dietary targets (≥80%) Intermediate: 2 or 3 dietary targets (40%–79%) Poor: <2 dietary targets (<40%) | Sum of scores for primary metrics | 0 (worst) to 100 (best)[§](https://www-ahajournals-org.proxy.libraries.rutgers.edu/reader/content/10.1161/CIR.0000000000000757/format/epub/EPUB/xhtml/index.xhtml#tab5-1fn5) Ideal: 80–100 Intermediate: 40–79 Poor: <40 |
| AHA Diet Score (secondary) | Ideal: 4 or 5 dietary targets (≥80%) Intermediate: 2 or 3 dietary targets (40%–79%) Poor: <2 dietary targets (<40%) | Sum of scores for primary and secondary metrics | 0 (worst) to 100 (best)[§](https://www-ahajournals-org.proxy.libraries.rutgers.edu/reader/content/10.1161/CIR.0000000000000757/format/epub/EPUB/xhtml/index.xhtml#tab5-1fn5) Ideal: 80–100 Intermediate: 40–79 Poor: <40 |

AHA indicates American Heart Association; and SSBs, sugar-sweetened beverages.

* Consistent with other dietary pattern scores, the highest score (10) was given for meeting or exceeding the AHA target (eg, at least 4.5 cups of fruit and vegetables per day; no more than 1500 mg/d of sodium), and the lowest score (0) was given for zero intake (protective factors) or for very high intake (harmful factors). The score for each metric was scaled continuously within this range. For harmful factors, the level of high intake that corresponded to a zero score was identified as approximately the 90th percentile distribution of US population intake.

† Selected by the AHA based on evidence for likely causal effects on cardiovascular events, diabetes mellitus, or obesity; a general prioritization of food rather than nutrient metrics; consistency with US and AHA dietary guidelines; ability to measure and track these metrics in the US population; and parsimony, that is, the inclusion of as few components as possible that had minimal overlap with each other while at the same time having some overlap with the many other relevant dietary factors that were not included.[^2^](https://www-ahajournals-org.proxy.libraries.rutgers.edu/reader/content/10.1161/CIR.0000000000000757/format/epub/EPUB/xhtml/index.xhtml#R5-2) The AHA dietary metrics should be targeted in the context of a healthy diet pattern that is appropriate in energy balance and consistent with a DASH (Dietary Approaches to Stop Hypertension)-type eating plan, including but not limited to these metrics.

‡ Including up to one 8-oz serving per day of 100% fruit juice and up to 0.42 cups/d (3 cups/wk) of starchy vegetables such as potatoes or corn.

§ The natural range of the primary AHA Diet Score is 0 to 50 (5 components), and the natural range of the secondary AHA Diet Score is 0 to 80 (8 components). Both scores are then rescaled to a range of 0 to 100 for comparison purposes. The ideal range of the primary AHA Diet Score corresponds to the AHA scoring system of meeting at least 4 of 5 binary dietary targets (≥80%), the intermediate range corresponds to meeting 2 or 3 dietary targets (40%–79%), and the poor range corresponds to meeting <2 dietary targets (<40%). The same ranges are used for the secondary AHA Diet Score for consistency and comparison.

## eTable 2. Demographic Characteristics, Comorbidities, and Lifestyle Factors based on Perceived Diet Quality Among Subgroup Population with Poor Measured Diet Quality

|  |  |  | **Perceived Diet Quality** | |
| --- | --- | --- | --- | --- |
|  | **Missing (n)** | **Overall** | **Not Healthy** | **Healthy** |
| Total population (n)^a^ |  | 14,952 | 11,547 | 3,405 |
| **Sociodemographics** |  |  |  |  |
| Age | 0 | 45 (16) | 44 (16) | 49 (17) |
| Sex | 0 |  |  |  |
| Male |  | 7,895 (53%) | 5,556 (47%) | 1,501 (48%) |
| Female |  | 7,057 (47%) | 5,991 (53%) | 1,904 (52%) |
| Race/Ethnicity | 0 |  |  |  |
| Non-Hispanic White |  | 6,348 (66%) | 4,749 (65%) | 1,599 (71%) |
| Hispanic |  | 3,995 (15%) | 3,314 (17%) | 681 (10%) |
| Non-Hispanic Black |  | 3,313 (12%) | 2,618 (13%) | 695 (10%) |
| Other |  | 1,296 (6.5%) | 866 (5.8%) | 430 (8.6%) |
| Education | 9 |  |  |  |
| Less than High school |  | 3,928 (18%) | 3,148 (19%) | 780 (14%) |
| High school graduate |  | 3,707 (26%) | 2,976 (27%) | 731 (23%) |
| Some college |  | 4,589 (33%) | 3,577 (34%) | 1,012 (30%) |
| College graduate or above |  | 2,719 (23%) | 1,838 (21%) | 881 (32%) |
| Household income | 726 |  |  |  |
| Less than $20,000 |  | 3,213 (16%) | 2,524 (16%) | 689 (14%) |
| $20,000-$75,000 |  | 7,685 (51%) | 6,017 (52%) | 1,668 (49%) |
| Greater than $75,000 |  | 3,328 (33%) | 2,441 (32%) | 887 (37%) |
| Interview language | 0 |  |  |  |
| English |  | 12,928 (93%) | 9,802 (92%) | 3,126 (97%) |
| Spanish |  | 2,024 (6.7%) | 1,745 (7.7%) | 279 (3.4%) |
| Poverty income ratio | 1,173 | 2.84 (1.65) | 2.76 (1.64) | 3.10 (1.65) |
| **Comorbidities** |  |  |  |  |
| Congestive heart failure | 0 | 408 (1.9%) | 300 (1.8%) | 108 (2.2%) |
| Coronary artery disease | 0 | 508 (2.6%) | 362 (2.4%) | 146 (3.6%) |
| Myocardial infarction | 0 | 568 (2.8%) | 410 (2.6%) | 158 (3.4%) |
| Stroke | 0 | 536 (2.7%) | 398 (2.6%) | 138 (2.9%) |
| Emphysema | 19 | 339 (1.9%) | 259 (1.9%) | 80 (2.1%) |
| Cancer | 17 | 1,197 (8.4%) | 822 (7.7%) | 375 (11%) |
| Diabetes mellitus | 309 | 1,559 (7.4%) | 1,368 (8.6%) | 322 (6.6%) |
| Hypertension | 22 | 4,925 (29%) | 3,861 (30%) | 1,097 (28%) |
| High cholesterol | 2,296 | 4,554 (35%) | 3,824 (37%) | 1,167 (39%) |
| Depression | 6,618 | 2,224 (26%) | 1,900 (29%) | 324 (17%) |
| BMI | 124 | 29 (7) | 30 (7) | 27 (6) |
| Waist circumference (cm) | 437 | 100 (17) | 101 (17) | 95 (15) |
| Triglyceride (mg/dL) | 748 | 159 (121) | 164 (125) | 142 (103) |
| **Lifestyle** |  |  |  |  |
| Alcoholic drinks per day | 0 | 0.22 (0.63) | 0.20 (0.60) | 0.28 (0.73) |
| Moderate activity | 34 | 5,667 (44%) | 4,119 (41%) | 1,548 (52%) |
| Vigorous activity | 44 | 3,339 (26%) | 2,381 (24%) | 958 (34%) |
| Smoking | 7 | 7,317 (49%) | 5,682 (50%) | 1,635 (46%) |
| Dietary guidelines awareness | 4,544 | 2,755 (32%) | 2,100 (31%) | 655 (34%) |
| AHA primary score (standardized) | 0 | 28 (8) | 28 (8) | 29 (8) |
| AHA secondary score (standardized) | 0 | 34 (11) | 34 (11) | 36 (11) |

Healthy perceived diet quality defined as “Very Good” and “Excellent” responses; Not Healthy perceived diet quality: “Poor”, “Fair”, “Good” responses.

^a^ Categorical variables are reported with unweighted counts and survey-weighted percentages. Continuous variables are reported with mean and standard deviation.

## eTable 3. Demographic Characteristics, Comorbidities, and Lifestyle Factors based on Perceived Diet Quality Among Subgroup Population in Sensitivity Analysis

|  |  |  | **Perceived Diet Quality** | |
| --- | --- | --- | --- | --- |
|  | **Missing (n)** | **Overall** | **Not Healthy** | **Healthy** |
| Total population (n) |  | 14,952 | 5,332 | 9,620 |
| **Sociodemographics** |  |  |  |  |
| Age | 0 | 45 (16) | 42 (15) | 46 (17) |
| Sex | 0 |  |  |  |
| Male |  | 7,895 (53%) | 5,142 (52%) | 2,752 (53%) |
| Female |  | 7,057 (47%) | 2,580 (47%) | 4,477 (48%) |
| Race/Ethnicity | 0 |  |  |  |
| Non-Hispanic White |  | 6,348 (66%) | 1,999 (60%) | 4,349 (69%) |
| Hispanic |  | 3,995 (15%) | 1,717 (20%) | 2,278 (13%) |
| Non-Hispanic Black |  | 3,313 (12%) | 1,309 (15%) | 2,004 (11%) |
| Other |  | 1,296 (6.5%) | 307 (5.2%) | 989 (7.1%) |
| Education | 9 |  |  |  |
| Less than High school |  | 3,928 (18%) | 1,687 (23%) | 2,241 (15%) |
| High school graduate |  | 3,707 (26%) | 1,417 (28%) | 2,290 (25%) |
| Some college |  | 4,589 (33%) | 1,597 (33%) | 2,992 (33%) |
| College graduate or above |  | 2,719 (23%) | 630 (16%) | 2,089 (27%) |
| Household income | 726 |  |  |  |
| Less than $20,000 |  | 3,213 (16%) | 1,292 (19%) | 1,921 (14%) |
| $20,000-$75,000 |  | 7,685 (51%) | 2,824 (54%) | 4,861 (50%) |
| Greater than $75,000 |  | 3,328 (33%) | 942 (27%) | 2,386 (36%) |
| Interview language | 0 |  |  |  |
| English |  | 12,928 (93%) | 4,336 (90%) | 8,592 (95%) |
| Spanish |  | 2,024 (6.7%) | 996 (10%) | 1,028 (5.0%) |
| Poverty income ratio | 1,173 | 2.84 (1.65) | 2.55 (1.61) | 2.99 (1.65) |
| **Comorbidities** |  |  |  |  |
| Congestive heart failure | 0 | 408 (1.9%) | 148 (1.8%) | 260 (1.9%) |
| Coronary artery disease | 0 | 508 (2.6%) | 173 (2.4%) | 335 (2.8%) |
| Myocardial infarction | 0 | 568 (2.8%) | 206 (2.8%) | 362 (2.8%) |
| Stroke | 0 | 536 (2.7%) | 199 (3.2%) | 337 (2.4%) |
| Emphysema | 19 | 339 (1.9%) | 131 (2.2%) | 208 (1.8%) |
| Cancer | 17 | 1,197 (8.4%) | 341 (7.2%) | 856 (9.1%) |
| Diabetes mellitus | 256 | 1,690 (8.1%) | 702 (10%) | 988 (7.1%) |
| Hypertension | 22 | 4,958 (30%) | 1,844 (32%) | 3,114 (28%) |
| High cholesterol | 2,262 | 4,991 (37%) | 1,754 (37%) | 3,237 (38%) |
| Depression | 6,618 | 2,224 (26%) | 1,119 (36%) | 1,105 (21%) |
| BMI | 124 | 29 (7) | 31 (8) | 28 (6) |
| Waist circumference (cm) | 437 | 100 (17) | 103 (18) | 98 (16) |
| Triglyceride (mg/dL) | 748 | 159 (121) | 170 (132) | 154 (114) |
| **Lifestyle** |  |  |  |  |
| Alcoholic drinks per day | 0 | 0.22 (0.63) | 0.20 (0.59) | 0.23 (0.66) |
| Moderate activity | 34 | 5,667 (44%) | 1,669 (35%) | 3,998 (48%) |
| Vigorous activity | 44 | 3,339 (26%) | 940 (19%) | 2,399 (30%) |
| Smoking | 7 | 7,317 (49%) | 2,791 (54%) | 4,526 (46%) |
| Dietary guidelines awareness | 4,544 | 2,755 (32%) | 884 (27%) | 1,871 (34%) |
| AHA primary score (scaled) | 1 | 47 (10) | 46 (10) | 48 (11) |
| AHA secondary score (scaled) | 0 | 28 (8) | 27 (9) | 29 (8) |

Healthy perceived diet quality defined as “Good”, “Very Good”, and “Excellent” responses; Not Healthy perceived diet quality: “Poor” and “Fair” responses.

^a^ Categorical variables are reported with unweighted counts and survey-weighted percentages. Continuous variables are reported with mean and standard deviation.

## eTable 4. Risk Ratios of Perceiving Poor Diet Quality as Healthy Among Subgroup Population in Sensitivity Analysis

|  | **Univariate** | **Multivariable** |
| --- | --- | --- |
| **Variable** | **Risk Ratio (95% CI)** | **Risk Ratio (95% CI)** |
| Age group^1^ |  |  |
| 35-49 | 1.02 (0.97, 1.07) | **1.10 (1.04, 1.16)** |
| 50-64 | **1.12 (1.07, 1.18)** | **1.30 (1.23, 1.39)** |
| 65+ | **1.29 (1.23, 1.35)** | **1.52 (1.43, 1.62)** |
| Sex^2^ |  |  |
| Male | 0.98 (0.95, 1.02) | **0.95 (0.92, 0.99)** |
| Race/Ethnicity^3^ |  |  |
| Hispanic | **0.81 (0.78, 0.85)** | **0.88 (0.84, 0.93)** |
| Non-Hispanic Black | **0.85 (0.82, 0.89)** | **0.89 (0.85, 0.93)** |
| Other | 1.05 (0.99, 1.11) | 1.03 (0.96, 1.10) |
| Education^4^ | **1.24 (1.19, 1.28)** | **1.10 (1.05, 1.16)** |
| Household income^5^ | **1.15 (1.11, 1.19)** | **1.06 (1.02, 1.10)** |
| Comorbidities |  |  |
| Myocardial infarction | 1.01 (0.94, 1.08) | 1.02 (0.94, 1.11) |
| Diabetes mellitus | **0.87 (0.81, 0.92)** | **0.90 (0.84, 0.97)** |
| Hypertension | **0.95 (0.91, 0.98)** | **0.93 (0.89, 0.97)** |
| High cholesterol | 1.01 (0.98, 1.05) | 0.98 (0.94, 1.01) |
| High risk waist circumference^6^ | **0.85 (0.82, 0.87)** | **0.92 (0.88, 0.96)** |
| BMI^7^ |  |  |
| Obesity | **0.77 (0.74, 0.80)** | **0.87 (0.82, 0.93)** |
| Overweight | **0.95 (0.92, 0.98)** | 0.98 (0.94, 1.01) |
| Underweight | **0.78 (0.66, 0.92)** | 0.81 (0.66, 1.00) |
| Above moderate alcoholic drinks per day^8^ | 1.02 (0.94, 1.10) | 1.01 (0.93, 1.09) |
| Vigorous activity^9^ | **1.20 (1.16, 1.24)** | **1.19 (1.15, 1.23)** |
| Smoking^10^ | **0.90 (0.87, 0.92)** | **0.92 (0.88, 0.96)** |

Healthy perceived diet quality defined as “good”, “very good”, and “excellent” responses to “How healthy is your diet?” ^1^ Reference range: Age group 20-34; ^2^ Reference range: Female; ^3^ Reference range: White and Other includes non-Hispanic Asian and multiracial; ^4^ Ordered variable: Less than High School, High school graduate, Some college, College graduate or above; ^5^ Ordered variable: Less than $20K, $20-75K, $75K+; ^6^ Reference range: Low risk waist circumference; ^7^ Reference range: Healthy; ^8^ Reference range: Moderate alcoholic drinks per day; ^9^ At least 10 continuous minutes; ^10^ At least 100 cigarettes in lifetime
